# 14.3 CAUSES AND PREVENTION OF AGGRESSION FROM PSYCHOTIC INPATIENTS

**DOI:** 10.1093/schbul/sby014.056

**Published:** 2018-04-01

**Authors:** Henk Nijman

**Affiliations:** Radboud University Nijmegen

## Abstract

**Background:**

Patients with schizophrenia and other psychotic disorders have an increased likelihood of engaging in violent behavior. These increased risks of dangerous and aggressive behavior, in combination with a lack of insight in their own illness, relatively often make involuntary admission of acutely disturbed psychotic patients on locked psychiatric admissions wards often inevitable. On these locked psychiatric admissions wards, aggression from psychotic patients against staff and fellow patients is a prevalent phenomenon, with the mean in the Netherlands being about 18 aggressive incidents per bed per year on locked psychiatric admissions wards.

**Methods:**

In the lecture, a model of what causes or triggers aggressive behavior on (locked) psychiatric wards is presented. In this model, patient, ward and staff variables are integrated to explain why, and in what specific situations, psychotic patients particularly run a high risk of engaging in aggressive behavior.

**Results:**

Based on the presented model, a number of preventive measures can be formulated.

On the patient level, the administration of anti-psychotic medication is used to reduce the negative cognitive schemes and delusional thoughts that are depicted in the center of the model. A more novel intervention at the patient level may be the additional administration of nutritional supplements with (among others) high levels of omega 3 fatty acids. The results of two Dutch studies on this topic will be briefly presented in the lecture, among which a RCT on the effects of the use of nutritional supplements on aggressiveness.

On the staff level, the use of short-term (daily) risks assessments by the ward nursing staff, among others by means of the six item BrØset Violence Checklist (BVC), has been found to reduce aggressiveness and the use of coercive measures on psychiatric wards in two cluster randomized RCTs.

On the ward level, studies indicate that aggression on psychiatric wards can be reduced by preventing overcrowding on psychiatric wards, and by providing more space and privacy to the patients.

**Discussion:**

The proposed model elucidates how certain patient, staff and ward characteristics may interact in causing aggression. The model also emphasizes that repeated inpatient aggression may be the result of a vicious circle, i.e. inpatient violence is often followed by an increase in environmental and/or communication stress on the patient, thereby heightening the risk of a repeated outburst of violence.

